# The association between physical symptoms and depression among medical students in Bahrain

**DOI:** 10.5116/ijme.5a2d.16a3

**Published:** 2017-12-15

**Authors:** Ammar M.Y. Abdelaziz, Khalid T. Alotaibi, Jarah H. Alhurayyis, Turky A. Alqahtani, Aamer M. Alghamlas, Haifa M. Algahtani, Haitham A. Jahrami

**Affiliations:** 1 College of Medicine and Medical Sciences, Kingdom of Bahrain

**Keywords:** Depression, physical symptoms, medical students, Bahrain

## Abstract

**Objectives:**

To examine the association between depression and physical symptoms among medical students in Bahrain.

**Methods:**

The present study employed a cross-sectional design.  A total of 160 students were recruited, 41.3% were male and 58.8% female, using a convenience sampling approach. Participants completed the validated Patients Health Questionnaires (PHQs) in which they provided information about demographics, physical symptoms, and depression. Results were considered significant if p <0.05.

**Results:**

Nearly nineteen percent of the participants have moderate to severe depression, and 42.2% has moderate to severe physical symptoms.  Participants reported different physical symptoms, sleep problems, 40%; lethargy, 31.9%; and headaches, 23.8%. The results of the logistic regression showed that there was a significant association between age and gender (χ^2^_(3)_ = 32.28, p < 0.001). Sleep and gastrointestinal symptoms were the most associated with depression, respectively (χ^2^_(3)_=49.77, p<0.001) and (χ^2^_(3)_=49.77, p< 0.05).

**Conclusions:**

The association between depression and physical symptoms are considerably high among medical students in Bahrain.  Medical educators should take such symptoms seriously among medical students as it may have serious consequences on the mental health of medical students. In practice, adequate awareness initiatives should be organized and provided for medical students to help them overcome their challenges they face. Additionally, incorporating screening self-screening strategies in the medical curriculum can be beneficial for early detections of mental health problems. The Implications and limitations of the study are discussed.

## Introduction

Medical education is associated with a considerable level of stress thereby its negative effects on the health of students has been addressed in the literature.^1^ Several studies have pointed that the source of stress stems from three main areas: academic, social and financial strains.^2^^,^^3^ In their pre-clinical year's medical students endure a stressful competitive environment with multiple demands and challenges.^2^ Dealing with overloaded medical curriculum, competing with peers, being away from home and meeting high expectations imposed by parents and society to excel are among the common stressful transitions at this stage.^3^ As they progress to clinical years the contact with patients, diseases and death, graduation and acquiring an internship or residency position adds more stress to achieving targets.^3^ The combination of all of these stressors can predispose medical students to a variety of psychological disorders including depression, anxiety, somatic symptoms, and even suicidal ideation.^1^^,^^4^ Depression is the most common experience with reported rates being as high as 28%.^4^^-^^8^ The effects of depression on mental wellbeing and physical health are also documented.^7^^,^^8^ Depression can significantly decrease student's performance and productivity and negatively affect interpersonal relationships, and if left untreated it might lead to long-term impairments in professional performance and social skills. ^9^^-^^12^ Depression among medical students can often go undiagnosed for medical students, as it can be confused with stress resulting from increased assignments or exams, or the pressure of social interaction with new colleagues and patients.^11^^,^^12^ Physical symptoms are very common in depressive disorders and these symptoms may lead to the development of chronic pain and may complicate treatment.^10^^,^^13^

Kroenke and colleagues studied 1000 participants from the general population and found that the number of physical symptoms present were highly predictive of depression and associated with functional impairment.^14^ Participants who reported one physical symptom had a 2% rate of depression, compared with a rate of 60% of depression in participants who reported nine or more symptoms.^14^ The higher the severity of physical symptoms is associated with the more severe depressive disorder and higher functional impairment.^14^^,^^15^

Over the years an enormous amount of research has been carried out in an attempt to examine the association between physical symptoms and depression. Most of these studies have been conducted in Western Europe, North America and Australia. However, few attempts have been made to investigate the association between physical symptoms and depression in Arabian counties, particularly in Bahrain. Therefore, the aim of this study is to examine the association between physical symptoms and depression among medical students in Bahrain. Providing evidence of such an association may encourage Bahraini medical educators to provide pastoral support for medical students, especially those who have mental health issues.

## Methods

### Participants

One hundred and sixty students studying medicine were recruited for this study, 41.3% were male and 58.8% female. The age of participants ranged from 18 to 26 years (M= 21.5, SD=2.3).

### Instrument and procedures

The Patients Health Questionnaires (PHQs) are set of diagnostic tools used to recognize mental health disorders in convenient and reliable manner.^15^ The PHQs were developed in the mid-1990s at Columbia University and contained 12 different mental health disorders. In our research, we used the PHQ-15 module to measure the physical symptoms and the PHQ-9 module to measure depressive disorders.

The data-collection tool contained short basic demographic data, and two short validated self-administered questionnaires. The PHQ-15 is a self-administered questionnaire developed for detection of a list of 15 somatic symptoms. Each of the 15 symptoms is scored on a three-point Likert scale (Not bothered at all = 0 Bothered a little = 1 Bothered a lot = 2). The PHQ-15 overall score was interpreted as follows (0-4 minimal somatic symptoms, 5-9 low somatic symptoms, 10-14 medium somatic symptoms, 15 or more high somatic symptoms).^15^^,^^16^ Increasing scores on the PHQ-15 are strongly associated with functional impairment, disability, and healthcare utilization.

The PHQ-9 is the depression module, which scores each of the nine DSM-IV criteria on a four-point Likert scale (0=not at all to 3=nearly every day). It is used to monitor the presence of depression rather than being a screening tool. PHQ-9 classification of symptoms is (5-9 minimal symptoms, 10-14 minor depression, 15-19 moderate major depression, >20 severe major depression).^17^

The psychometric properties of the PHQ-15 and PHQ-9 were established in clinical settings in different cultures.^16^^,^^18^^,^^19^ Results from validation studies showed an excellent validity and test re-test reliability. The data-collection instruments also demonstrated high sensitivity and specificity. In our research, the original English language tools were used to preserve their psychometric properties, and because the participants are comfortable with English terminology.

This study was approved by the research ethic committee of College of Medicine and Medical Sciences, Kingdom of Bahrain. Participation in the study was fully voluntary. The anonymity of the participants was maintained across the study. We asked students not to write their names on the questionnaire, and even we did not use any identification during the data collection process.

### Data analysis

Data were analyzed using STATA 13.1. Descriptive statistics (M mean, and SD Standard Deviation) and frequency counts were used to describe the participants' demographics and to describe physical and depressive symptoms and severity. Logistic regression was used for predicting the binary outcome variable (depression) using a number of physical symptoms controlling for age and sex. Logistic regression was performed for predicting the binary outcome variable (depression) using the fifteen different physical symptoms.

## Results

### Depression

Overall, 15 % of medical students had minor depression, 11.3% had moderate depression, and 7.7% had severe depression. No depression reported among 34.4% of students. The results showed that 34.4% of medical students had no depression, but 31.9% of students reported minor depression. symptoms.

### Physical symptoms

The results showed that approximately 20% of medical students had no or minimal insignificant physical symptoms, but 35% had low physical symptoms, 25% had moderate physical symptoms, and 20% had severe physical symptoms complaints. Medical students have reported a mean of physical symptoms (M 7.2, SD 3.4). Table 1 shows the seven most common symptoms experienced by all medical students. These are sleep problems, lethargy, headaches, back pain, stomach pain, joints pain and nausea, gas or indigestion. The most severe symptom experienced was sleep problems.

### Association between physical symptoms and depression

Logistic regression models were used to examine whether the number and type of physical symptoms reported by medical students are predictive of depression disorders. The first model included depression as an outcome variable and the number of physical symptoms age and sex as independent variables. Age and sex were included in the model to control for their confounding effect. As we can see from Table 2, the number of physical symptoms considered as a strong predictor of the development of depression symptoms.

**Table 1:** Physical symptoms of medical students (N=160)

Physical Symptom	Not bothered	Bothered a little	Bothered a lot
	n (%)	n (%)	n (%)
Stomach pain	69 (41.1)	74 (46.9)	16 (10)
Back pain	65 (40.6)	68 (42.5)	27 (16.9)
Pain in your arms, legs, or joints (knees, hips, etc.)	66 (41.3)	69 (43.1)	25 (15.6)
Feeling tired or having little energy	41 (25.6)	68 (42.5)	51 (31.9)
Trouble falling or staying asleep, or sleeping too much	40 (25)	56 (35)	64 (40)
Menstrual cramps or other problems with your periods	80 (50)	54 (33.8)	26 (16.3)
Pain or problems during sexual intercourse	142 (88.8)	9 (5.6)	9 (5.6)
Headaches	50 (31.3)	72 (45)	38 (23.8)
Chest pain	107 (66.9)	38 (23.8)	15 (9.4)
Dizziness	96 (60)	49 (30.6)	15 (9.4)
Fainting spells	136 (85)	20 (12.5)	4 (2.5)
Feeling your heart pound or race	89 (55.6)	55 (34.4)	16 (10)
Shortness of breath	94 (58.8)	55 (34.4)	11 (6.9)
Constipation, loose bowels, or diarrhea	80 (50)	56 (35)	24 (15)
Nausea, gas, or indigestion	83 (51.9)	55 (34.4)	22 (13.8)

A second logistic regression model was computed using the depression as an outcome variable and the list of physical symptoms as independent or explanatory variables. Table 3 shows the odds ratios instead of regression coefficients because they serve as a more intuitive approach to report. The most commonly associated symptoms were "trouble falling or staying asleep, or sleeping too much", "nausea, gas, or indigestion", "stomach pain", "pain or problems during sexual intercourse", "back pain", "headaches", "chest pain", and "feeling your heart pound or race". However, only "trouble falling or staying asleep, or sleeping too much", and gastrointestinal "nausea, gas, or indigestion" reached statistical significance as predictors.

## Discussion

This study is one of the few studies done in the region on the topic of physical symptoms and depression and its implications for medical schools. Our results, i.e. nearly 19% of students had moderate to severe depression, and 46.2% had moderate to severe physical symptoms, are consistent with previous results.^4^^,^^6^^,^^8^^,^^13^ However, these studies were not conducted in medical schools. Therefore, further research will have to shed light on physical symptoms in Arabian countries.

**Table 2:** Logistic regression of Depression as outcome and the number of physical symptoms (N=160)

Depression (Outcome)	Coefficient	Std. Err.	z	P	95%CI	
Number of physical symptoms	0.34	0.07	4.84	0.001	0.20	0.50
Gender	-0.34	0.41	-0.84	0.40	-1.1	0.46
Age	0.07	0.08	0.98	0.33	-0.08	0.24
Constant	-4.51	2.06	-2.19	0.03	-8.6	-0.46

Our results indicate that depression symptoms among medical students were significantly lower than physical symptoms. A possible interpretation of this finding is that students were more comfortable to report physical symptoms than that of mood symptoms. The implication of this finding is that medical educators can detect early symptoms of depression in students. When available, they are well positioned to provide primary and secondary prevention to students who have symptoms of depression.

**Table 3:** Logistic regression of depression as outcome and the list of physical symptoms in the PHQ-15 (N=160)

Depression (Outcome)	Odds Ratio		Std. Err.	z	P		95%CI	
Stomach pain	2.55	1.30		1.84		0.07	0.94	6.94
Back pain	1.53	0.73		0.89		0.37	0.60	3.88
Pain in your arms, legs, or joints (knees, hips, etc.)	0.83	0.37		-0.42		0.67	0.35	1.99
Feeling tired or having little energy	1.41	0.78		0.61		0.54	0.47	4.20
Trouble falling or staying asleep, or sleeping too much	9.28	7.38		2.80		0.01*	1.95	44.08
Menstrual cramps or other problems with your periods	0.86	0.39		-0.34		0.73	0.35	2.08
Pain or problems during sexual intercourse	2.16	1.39		1.19		0.23	0.61	7.61
Headaches	1.80	0.97		1.09		0.28	0.62	5.19
Chest pain	1.26	0.58		0.51		0.61	0.52	3.10
Dizziness	0.89	0.41		-0.25		0.80	0.36	2.19
Fainting spells	1.04	0.60		0.07		0.94	0.34	3.23
Feeling your heart pound or race	1.42	0.65		0.78		0.44	0.58	3.48
Shortness of breath	0.80	0.36		-0.50		0.62	0.33	1.93
Constipation, loose bowels, or diarrhea	0.98	0.44		-0.04		0.97	0.41	2.35
Nausea, gas, or indigestion	2.52	1.17		1.99		0.05*	1.02	6.28
Constant	0.01	0.01		-4.70		0.00	0.00	0.07

Another important finding was that the number of severe physical symptoms was a strong predictor of the development of depression symptoms. This finding is in agreement with the findings of Smith and colleagues which showed "the prevalence of a mood disorder in patients with 0 to 1, 2 to 3, 4 to 5, 6 to 8, and 9 or more symptoms was 2%, 12%, 23%, 44%, and 60%, respectively."^14^

Additional results indicate that sleep problems ("trouble falling or staying asleep, or sleeping too much") and gastrointestinal problems ("nausea, gas, or indigestion") make a significant contribution to the development of depression symptoms. This finding suggests that physical symptoms unexplained by a physiological process may be a strong predictor of the development of psychiatric disorders such as depression. However, further work is required to establish this.

Our study has several implications for medical education and practice. First, given there is a link between the number of physical symptoms and depression symptoms, and that students may only report physical symptoms, medical educators should take seriously multiple or unexplained physical complaints reported by students in order to prevent issues related to mental health. Therefore, some support systems should be available for students, e.g., confidential counselling services, student mental services and personal tutor. Second, the training of students and medical educators with respect to multiple or unexplained physical complaints and its relation to mental health issues may improve pastoral care and student support, which in turn may enhance the mental health and wellbeing of students.

### Limitations

Finally, a number of important limitations need to be considered. First, the nature of the self-administered questionnaire might introduce recall bias in remembering the presence of physical symptoms, and underreporting may have occurred due to students' reluctance to report depression symptoms. Second, this is a cross-sectional study and results were obtained at a specific point in time. Therefore, the results of the study might differ if the study were conducted at another time. Third, the study did not take into account the time in the academic year in which the study was conducted. This is important as exam times may hold increasing amounts of anxiety which may have changed the outcome to some extent. However, this was difficult to control because different academic years have their exams at different times. Finally, another major source of uncertainty is that the results are limited by one medical school in Bahrain meaning that our results are not generalizable. Finally, students were recruited using a convenience sampling approach, and hence sampling bias might distort the results of the study. Given these limitations, the results need to be interpreted cautiously.

## Conclusions

In this investigation, the aim was to examine the association between physical symptoms and depression among medical students. This study has shown that depression and physical symptoms are considerably high among medical students in Bahrain. Physical symptoms appear to be the first indication of depression; hence, such symptoms should be considered earlier and cannot be considered trivial. In practice, adequate awareness initiatives should be organized and provided for medical students to help them overcome their challenges they face. Additionally, incorporating screening self-screening strategies in the medical curriculum can be beneficial for early detections of mental health issues. Improving pastoral care and student mental health services are crucial in preventing of mental health issues. This research has thrown up many questions in need of further investigation to gain a greater understanding of mental health issues in Arabian countries.

## Conflict of Interest

The authors declare that they have no conflict of interest.
